# Epitypification with an emended description of *Tropidia connata* (Orchidaceae, Epidendroideae, Tropidieae)

**DOI:** 10.3897/phytokeys.80.12304

**Published:** 2017-06-05

**Authors:** Izai Alberto Bruno Sabino Kikuchi, Hirokazu Tsukaya

**Affiliations:** 1 Institute of Biology Leiden, Faculty of Science, Leiden University, Sylviusweg 72, 2333 BE, Leiden, Netherlands; 2 Botanical Gardens, Graduate School of Science, The University of Tokyo, 3-7-1 Hakusan, Bunkyo-ku, Tokyo 112-0001, Japan; 3 Department of Biological Sciences, Faculty of Science, The University of Tokyo, 7-3-1 Hongo, Bunkyo-ku, Tokyo 113-0033, Japan; 4 Bio-Next Project, Okazaki Institute for Integrative Bioscience, National Institutes of Natural Sciences, Yamate Build. #3, 5-1, Higashiyama, Myodaiji, Okazaki, Aichi 444-8787, Japan

**Keywords:** Borneo, emendation, epitypification, *Kalimantanorchis*, mycoheterotroph, Orchidaceae, *Tropidia*, Tropidieae

## Abstract

We found several specimens of *Tropidia connata*, a mycoheterotrophic orchid from Borneo, with features which have never been described in any of the existing literature, namely subterranean tubers. We mainly focus on the importance of the subterranean structures in comparison with the mycoheterotrophic genus *Kalimantanorchis* from the tribe Tropidieae. This finding of the tuberous structure gives a new insight into the classification of mycoheterotrohic species of Tropidieae and might affect the generic placement of *Kalimantanorchis*. We made a detailed study on the newly discovered specimens as well as the type, and found more diagnostic characters of *T. connata* than the previous description. Considering that the type specimen lacks the whole tuberous character, we consequently designate an epitype with a drawing and emend the description.

## Introduction

The genus *Tropidia* Lindl. (Orchidaceae, Epidendroideae, Tropidieae) currently contains about 30 species mainly distributed from Far East Asia, India, Sri Lanka, Indochina, Malaysia, Indonesia, the Philippines and New Guinea to northern Australia, spreading across the South Pacific Islands. There is one exception to this distribution: *Tropidia polystachya* (Sw.) Ames is native to Florida, the West Indies, Central America and northern South America including the Galapagos Islands (Chun-Kuei et al. 2013). *Tropidia* is often characterized by its woody stems with plicate leaves. However, two mycoheterotrophic species without any green leaves have been reported from this genus: *T. saprophytica* J.J.Sm. and *T. connata* J.J.Wood & A.L.Lamb.

Due to the rare occurrence of the mycoheterotrophic species in general and to the difficulty in extracting a complete root system, the subterranean parts of most mycoheterotrophic plants are less known than their aerial parts (Imhof et al. 2013). This is also the case for *Tropidia connata*, with no description provided for its subterranean parts (Wood and Cribb 1994). However, in 2011 and 2012, multiple specimens of *T. connata* with underground tuberous structure were collected by the last author from the heart of Borneo, inside the Betung-Kerihun National Park, (West Kalimantan, Indonesia), where many new mycoheterotrophic taxa were discovered (Tsukaya and Okada 2005, 2012a, b, c, 2013a, b, Tsukaya et al. 2011). In addition to the subterranean structures, we also found some remarkable floral features, which were also not described in the original description (Wood and Cribb 1994). As the type specimen lacks the tuber structure, we therefore chose one of the newly collected specimens to designate as an epitype with a drawing. We consequently provide an emended description of this enigmatic species, *T. connata*.

## Materials and methods

We collected three specimens from three localities of Indonesia under permission of Secretariat of Permission for Foreign Research, The Ministry of Research and Technology, Republic of Indonesia (RISTEK). The specimens (two of which are with tubers) were examined in this study. We also examined the type specimen, A.Lamb, A.Surat & H.Lim 1512 (K000942868). The newly collected specimens were dried or kept in 50% (v/v) ethanol and deposited in both BO and TI (herbarium codes are according to the Index Herbariorum http://sweetgum.nybg.org/science/ih/). The type specimen is deposited in K. Morphological observation was carried out using a stereomicroscope (MZ16a; Leica Microsystems). All measurements were taken from dried and spirit herbarium collections and field notes. The measurements are presented as length × width, followed by units of measurements (mm or cm). The terminology used here is according to Tsukaya et al. (2011).

## Results

In the original description, the diagnostic characters of *Tropidia connata* are the fractiflex rachis, connate lateral sepals forming a synsepalum, and shortly spurred lip (Wood and Cribb 1994). We confirmed each of these features in both the type specimen and the newly collected specimens. However, we discovered three features other than these characters.

**Figure 1.**
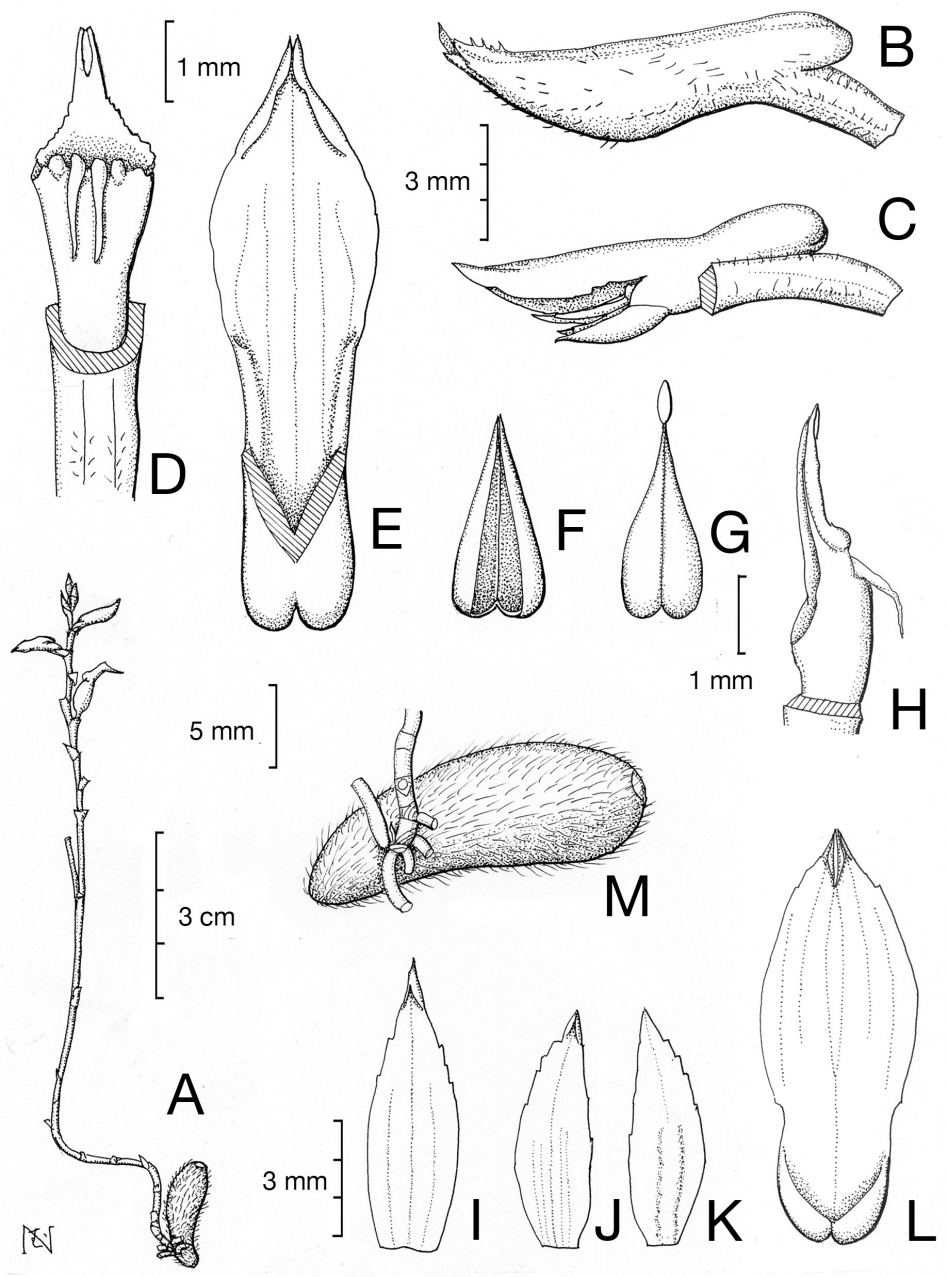
Drawing of *Tropidia connata* J.J.Wood & A.L.Lamb. **A** Gross morphology **B** Lateral view of unopened flower **C** Lateral view of the column with lip **D** Dorsal view of the column **E** Lip **F** Anther cap **G** Pollinia **H** Lateral view of the column **I** Dorsal sepal **J, K** Lateral petal of abaxial **(J**) and adaxial (**K**) side. **L** Lateral sepal **M** Tuber with basal part of inflorescence with short filamentous roots. Scales 3 cm (**A**), 3 mm (**B, C, I–K**), 1 mm (**D–H**), 5 mm (**M**). Drawing by Ms. Mutsuko Nakajima based on the specimen HT245.

**Figure 2.**
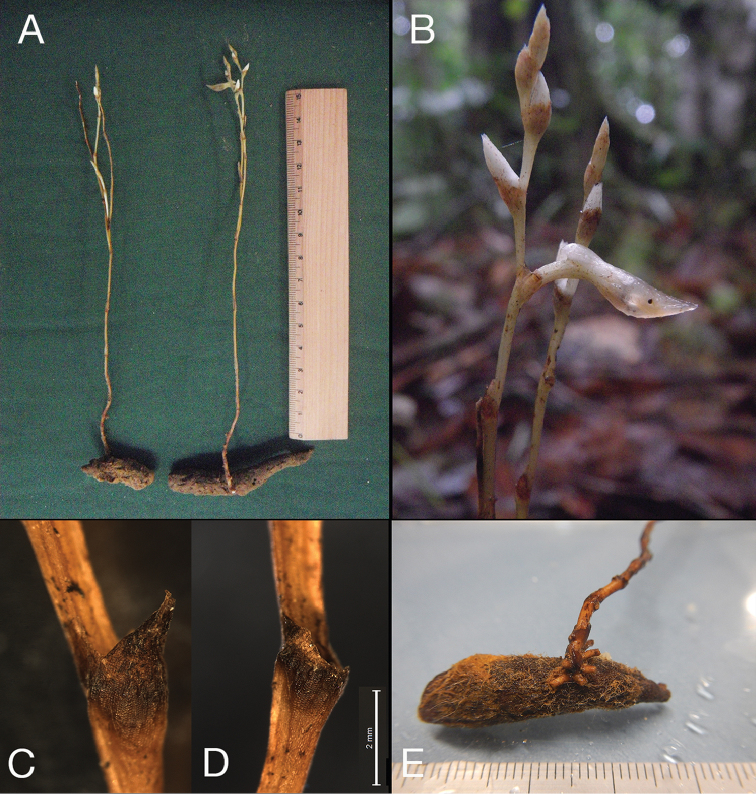
*Tropidia connata* J.J.Wood & A.L.Lamb. **A** Gross morphology of two individuals (specimen number 1040, collected in January 5, 2011). Scale in cm **B** Close-up of the inflorescence **C, D** Part of inflorescent stem with bracts showing before (**C**) and after (**D**) the detachment of the apical part at the abscission zonev **E** Subterrestrial part of the flowering individual. Divisions of scale in mm.

The most distinct feature that had not been described in the previous description is the subterranean tuberous structure (Figures 1M, 2A, E). The type specimen totally lacks this structure. The inflorescences occurred at the flanking region of the tuber, which was 2-7 cm long and 0.5-1 cm in diameter and covered with dense hairs (Figure 2E) (N = 4). One of the specimens (#1040) lacks a full length rhizome, but retains about 8 mm. Filamentous roots were poorly elongated in the specimen HT245 at the basal nodes of inflorescence (Figure 1M, Figure 2E), while most of the fibrous roots lack apices showing necrosis, suggesting the original length of the roots might be longer. On the other hand, we could not find any fibrous roots for the other specimens, suggesting that development of fibrous roots does not continue long, even if occurred.

The second distinct feature observed for the first time here is the two fiber-like protrusions attached to the adaxial side of the stigma (Figure 1D, H) (N = 2). Examination of the type specimen at the Kew Herbarium confirmed this structure as well. However, the color of this structure observed in the type was whitish and almost transparent, whereas the other floral specimens examined were black.

Another distinct feature recognized is the difference between the scale-like leaves and the flower bracts. While 5-10 scale-like leaves are seen on the stem and are ovate, acute, adpressed to the stem (N = 7) (Figure 1A, 2A), the bracts showed a distinct feature from scale-like leaves, the presence of an abscission layer 1-2 mm from the apex (N = 7) (Figure 2B-D). After maturation, the apical part falls away at this abscission layer.

We did not find any specimens with fully matured fruits, so we could not make an observation of the fruits or the seeds, which are also undescribed in the original description.

## Discussion

The tribe Tropidieae contains the genera *Tropidia*, *Corymborkis* Thouars, and the monotypic and mycoheterotrophic *Kalimantanorchis* Tsukaya, M.Nakaj. & H.Okada. *Corymborkis* does not contain any mycoheterotrophic species.

The presence of underground tubers has never been described in the genus *Tropidia*, although it was described for *Kalimantanorchis nagamasui* Tsukaya, M.Nakaj. & H.Okada (Tsukaya et al. 2011). Moreover, other leafy *Tropidia* species have fibrous roots and bear short and cylindrical tuber-like nodules at the distal end (Yeh et al. 2009, Chun-Kuei et al. 2013). Therefore, we hypothesize the tuber of *T. connata* may be equivalent to the tuber-like nodules of other leafy *Tropidia*. To confirm this hypothesis, we need comparative studies of the subterranean parts of both leafy and mycoheterotrophic *Tropidia*.

This discovery of the tuberous structure of *T. connata* clearly indicates that this is a shared feature of two of the mycoheterotrophic species in the tribe Tropidieae. However, the presence of a tuber has also never been reported for the other mycoheterotrophic species of Tropidieae, *T. saprophytica*. We consider that it is of great importance to discover new samples of *T. saprophytica* with well-preserved subterranean parts as well.

The main morphological feature of *Kalimantanorchis*, which made it distinct from other *Tropidia* mycoheterotrophic species at the time it was discovered, is the presence of the tubers. This result therefore raises the possibility that the genus *Kalimantanorchis* is not distinct from the genus *Tropidia* or that the tuber structure separately evolved in the tribe Tropidieae more than twice. The previous phylogenetic analyses of *Kalimantanorchis* (Tsukaya et al. 2011) did not include any samples of other mycoheterotrophic species of *Tropidia*, so we intend to perform further molecular phylogenetic analyses on both the samples of *T. connata* and *T. saprophytica*, as well as other leafy species of the tribe Tropidieae to obtain phylogenetic trees with better sampling, higher resolution, and higher support values than the previous work.

In addition to the tuberous structure, we found an abscission layer on bracts and two fibrous protrusions attached to the adaxial side of the stigma, both of which have never been described for other mycoheterotrophic Tropidieae species. The presence of such protrusions implies that there may have been some change in the pollination systems for *T. connata* due to the fully mycoheterotrophic habitat. Further research is needed to examine the function of these fibers in the pollination system to understand the possible pollination change in this species.

## Conclusions

The subterranean part of the type specimen was badly preserved as it lacks whole tuberous parts. Judging from the importance of this new finding of the tuberous structure, which provides possible changes in the classification of the tribe Tropidieae, we therefore consider it is necessary to choose one of the newly collected specimens (HT245) as an epitype. We designate the epitype, provide a drawing of the epitype, and emend the description of *Tropidia connata* at the end of this section as a taxonomic treatment.

Following this research, we conclude that the tribe Tropidieae is not thoroughly understood and little work has been done through either morphological or molecular methods. In particular, no comprehensive research of the whole genus *Tropidia* has been done, though this genus has a morphologically unique subterranean tuberous structure, and it seems that there still remain a lot of complications in this genus. It is also important to mention that several species have been discovered recently (Lin et al. 2006, Yeh et al. 2009, Chun-Kuei et al. 2013). We would like to continue and expand this study about the tribe Tropidieae focusing on *Tropidia* to contribute to a better understanding of this tribe.

### Taxonomic treatment

#### Tropidia connata

Taxon classificationPlantaeAsparagalesOrchidaceae

J.J.Wood & A.L.Lamb (1994: 47) emend. I.Kikuchi & Tsukaya

Figure 1

##### Type.

MALAYSIA. Borneo: Sabah, Sipitang District, Gunung Lumaku, 27 June 1992, A.Lamb, A.Surat, & H.Lim 1512 (holotype: K!, K000942868).

##### Epitype.

INDONESIA. West Kalimantan: Kabupaten Kapuas Hulu, Betung Kerihun National Park, en route from stream to ridge, a branch of Sungai (River) Sibau, 01°13.32'N; 113°06.2433'E, 240 m alt., 31 December 2011, H. Tsukaya, H. Okadada and A. Soejima HT245 (BO, TI), here designated.

##### Emended description.

Erect, mycoheterotrophic herb. Tubers 2–7×0.5–1 cm, dark brown, pubescent. Roots 2–5 mm long, short, occurring at the basal 1–3 nodes of stem. Stem 10–22 cm high, ivory white to creamy colored, wiry, simple or branched, perennial, 1 mm thick, internodes 0.8–2.5 cm long, sparsely ramentaceous, bearing 5–10 ovate, acute, sheath-like leaves apressed to the stem, 3–5 mm long. Inflorescence 5–15 flowered, 1–2 flowers open at a time; rachis 2–9 cm long, creamy white, internodes 4–10 mm, slightly zigzag, sparsely ramentaceous; floral bracts 3–5 mm long, off white tipped brown, triangular, acute to acuminate, with a abscission zone at 1–2 mm from the apex, apex detached at the abscission zone after maturation, base of the bracts forming a sheath enclosing the stem. Flowers 1 cm across, non-resupinate, partially open, white, apex of the lip pale orange to yellow. Pedicel with ovary 3–7 mm long, white, sparsely ramentaceous. Dorsal sepal 5–5.9×1.5–1.8 mm, narrowly elliptic, acute, sparsely ramentaceous on the abaxial sides. Lateral sepals connate into a 4–7.4×2.5–2.7 mm ovate-elliptic, cymbiform synsepalum, apex minutely bifid. Petals 4.8–6×1.5 mm narrowly elliptic, acute and asymmetric with two keels at the adaxial surface. Lip 5–7.6×2–2.2 mm with 3 nerves, elliptic, acute, concave, margin minutely erose, curving back to a horizontal position, spur saccate, obtuse, bifid, enclosed by synsepalum, 1.1–4×1 mm. Column 3–3.9×1.6–1.9 mm, ovate-trullate rostellum margin minutely erose 1.4–1.5 mm long, anther cap rostrate 2.5 mm long, stigma with calli-like protrusions and two fiber-like protrusions on the adaxial side of the column, the fibers 1.1–1.4 mm long. Pollinia 2, granular. Vicidium ellipsoid.

##### Additional specimens examined.

**INDONESIA. West Kalimantan**: Kabupaten Kapuas Hulu, Betung Kurihun National Park, Sungai (River) Menyakan, upstream of Sg. Sibau, 01°13.825'N; 113°03.963'E, 331m alt. to 01°13.82167'N; 113°03.9367'E, 331 m alt., 5 January 2011, H. Tsukaya, H. Okada and H. Nagamasu #1040 (BO, TI); Kabupaten Kapuas Hulu, Betung Kerihun National Park, on a ridge the junction of Sungai (River) Sibau and Sungai Minyakan, from 01°12.5567'N; 113°04.35'E, 298 m alt. to 01°12.325'N; 113°04.433'E, 341 m alt., 1 January 2012, H. Tsukaya, H. Okadada and A. Soejima HT250 (BO, TI).

## Supplementary Material

XML Treatment for Tropidia connata
